# Experimental study and modelling of asphaltene deposition on metal surfaces with superhydrophobic and low sliding angle inner coatings

**DOI:** 10.1038/s41598-021-95657-5

**Published:** 2021-08-19

**Authors:** Mohammad Haji-Savameri, Saeid Norouzi-Apourvari, Ahmad Irannejad, Abdolhossein Hemmati-Sarapardeh, Mahin Schaffie, Amir Mosavi

**Affiliations:** 1 Department of Petroleum Engineering, Shahid Bahonar University of Kerman, Kerman, Iran; 2 Department of Materials Engineering and Metallurgy, Shahid Bahonar University of Kerman, Kerman, Iran; 3 Institute of Research and Development, Duy Tan University, Da Nang, 550000 Vietnam; 4 Faculty of Environment and Chemical Engineering, Duy Tan University, Da Nang, 550000 Vietnam; 5 John Von Neumann Faculty of Informatics, Obuda University, Budapest, 1034 Hungary

**Keywords:** Energy science and technology, Engineering, Nanoscience and technology

## Abstract

Inner coatings have emerged as a novel technique to prevent the deposition of paraffin, wax, scale, and corrosion of pipelines during oil production and transport. Few studies addressed this technique for preventing asphaltene deposition. In this study, two superhydrophobic inner coatings, including polytetrafluoroethylene (PTFE) coating and nanosilica coating, were fabricated on metal surfaces and the asphaltene deposition on these coated surfaces was examined. A model oil solution was prepared using asphaltene and heptol and the effect of static and dynamic flow states on the amount of asphaltene deposition on uncoated electrodes, PTFE coated electrodes, and nanosilica coated electrodes were investigated. The results showed that the PTFE coating is more effective in reducing asphaltene deposition than nanosilica coating. The PTFE coating could reduce 56% of the deposition in a static state and more than 70% in a dynamic state at an asphaltene concentration of 2000 ppm. For PTFE coating in a dynamic state, the deposition rate is negligible in long times. In addition, it was found that the type of flow state affects the asphaltene deposition kinetics. The results demonstrate that, in the static state, the nth-order kinetics model, and in the dynamic state, the double exponential models are in best agreement with the experimental data.

## Introduction

One of the major challenges in the production and processing of crude oil is the deposition of heavy hydrocarbons including asphaltenes and waxes^1^ on the surface of well tubing, pipeline, and refining catalysts. Asphaltene molecules, as the heaviest and most polar components of crude oil^2^, are suspended in oil by resins under favorable conditions. Pressure, temperature, oil composition, the amount and type of injecting gas for enhanced oil recovery^3,4^, the amount of gas associated with oil, the type of flow in the porous media, and the characteristics of the fluid-containing pipes can be considered as effective factors on asphaltene precipitation and deposition^5^. Pressure is one of the most important parameters in the asphaltene deposition process, and other factors fall into the second order. The highest amount of asphaltene deposition occurs near the wellbore, where the highest pressure drop occurs with increasing production^6–8^. Reservoir pressure maintenance is probably the most effective technique to avoid asphaltene deposition in wells^9,10^. In oil production process, which is accompanied by a simultaneous decrease in pressure and temperature, asphaltene molecules precipitate and form sludge-like and highly adherent masses^11^. It is worth mentioning that the term deposition is commonly used to describe the process of precipitation^12^. Clarification of the differences between these two terms is important. The precipitation may be described as the formation of a solid phase from a liquid solution, while deposition can be defined as the formation and growth of a precipitated solid layer on the surface. Precipitation could be a prelude to deposition, but it does not necessarily guarantee deposition formation^13,14^. Asphaltene deposition could affect all components of the production system from the reservoir to the wellbore and up to the surface facility and pipelines. In porous media, the asphaltene deposition could plug the pore throats and alter the rock wettability^15^. The main mechanism(s) of deposition of asphaltene particles in porous media and resulting permeability damage have been investigated in the literature. In one of these studies, “surface deposition” was identified as the main mechanism in reducing the permeability of porous media^16^. It gradually blocks the wells and other transmission pipes and could also cause obstruction and failure of valves, separators, and other equipment it passes through. In refineries and petrochemical factories, even a small amount of asphaltene drastically reduces the efficiency of catalysts and other additives^17^.

The most costly and challenging problem related to depositions during crude oil production and processing cycle from reservoirs to petrochemicals and refineries occurs when access to the deposits is limited. The mitigation of deposition in the wellbore, for instance, requires chemical treatment which is costly and not an environmentally friendly method. Therefore, the deposition in the wellbore should be avoided as much as possible^18^. In tackling the challenge of asphaltene deposition in the industry, two main strategies are usually chosen, including deposition prevention and treatment and removal of deposition. The methods commonly used for deposition removal are generally divided into three categories, namely mechanical, chemical, and thermal methods^19^, among which the thermal methods are less efficient. Oil companies usually use chemicals, such as solvent (xylene) injection or acid injection using coil tubing, which can only remove depositions and are not capable to prevent the depositions buildup. In addition, the frequent need for solvent injections over a period of approximately 1 year (and in some cases months) will result in increased costs and repeated stoppages^20^. Therefore, prevention is always the preferred treatment if it is viable. In fact, chemical injection, and the use of mechanical and thermal methods, are normally introduced when the production process cannot be modified to prevent deposition^18^. The application of special coatings on the inner surfaces of pipes is another interesting approach to prevent or minimize deposition.

In the petroleum industry, the application of various coatings to prevent and reduce the deposition of solids such as asphaltene, scale, and wax, under flow conditions in wells and pipelines, has been investigated in various studies^21–29^. Coatings have been used for many years to prevent the deposition of paraffin, wax, scale, and corrosion of pipelines. Although their effectiveness in dealing with these cases has been approved, less effort has been devoted to asphaltene deposition. As mentioned earlier, asphaltene is the heaviest and most polar component of crude oil^2^, and water always competes with asphaltene molecules in adhering to different surfaces^30^. Therefore, the application of hydrophobic coatings could prevent the deposition of minerals and greatly reduce corrosion and asphaltene deposition.

Recent studies have tried to apply various coatings to prevent asphaltene deposition. Many of these methods are multi-stage and do not produce a surface with superhydrophobic properties^24^. More sophisticated coatings were also applied on different surfaces. The methods of fabrication and application of these coatings are very complex and require special equipment^21,27^. In addition, none of these coatings have superhydrophobic properties. The low sticking tendency of polytetrafluoroethylene (PTFE) polymers makes them desirable coating materials for controlling scales if applied appropriately. It should also be noted that the PTFE alone may have low surface energy and can create a hydrophobic surface, but what is important for reducing asphaltene deposition and other sediments is a low sliding angle, which can be achieved by creating the proper roughness on the surface.

The objective of this study is twofold. First, the effect of engineered superhydrophobic surfaces on reducing asphaltene deposition is examined, and later, the kinetic of asphaltene deposition on these coated surfaces is modeled. Asphaltene extracted from a crude oil sample and dissolved in heptol is subjected to an electric field for simulating the deposition process on coated and uncoated metal surfaces. Both PTFE-coated electrodes and nanosilica coated electrodes are examined. Different flow states and asphaltene concentrations of 2000, 1000, and 250 ppm are considered. These investigations could help us to better understand the factors affecting the kinetics of asphaltene deposition on coated and uncoated surfaces, which are necessary for choosing the optimal prohibitive measures in oil fields. It is necessary to mention that, the coatings fabricated in this study are suitable as inner coatings.

## Materials and experimental procedures

### Preparation of electrodes

**Figure 1:**
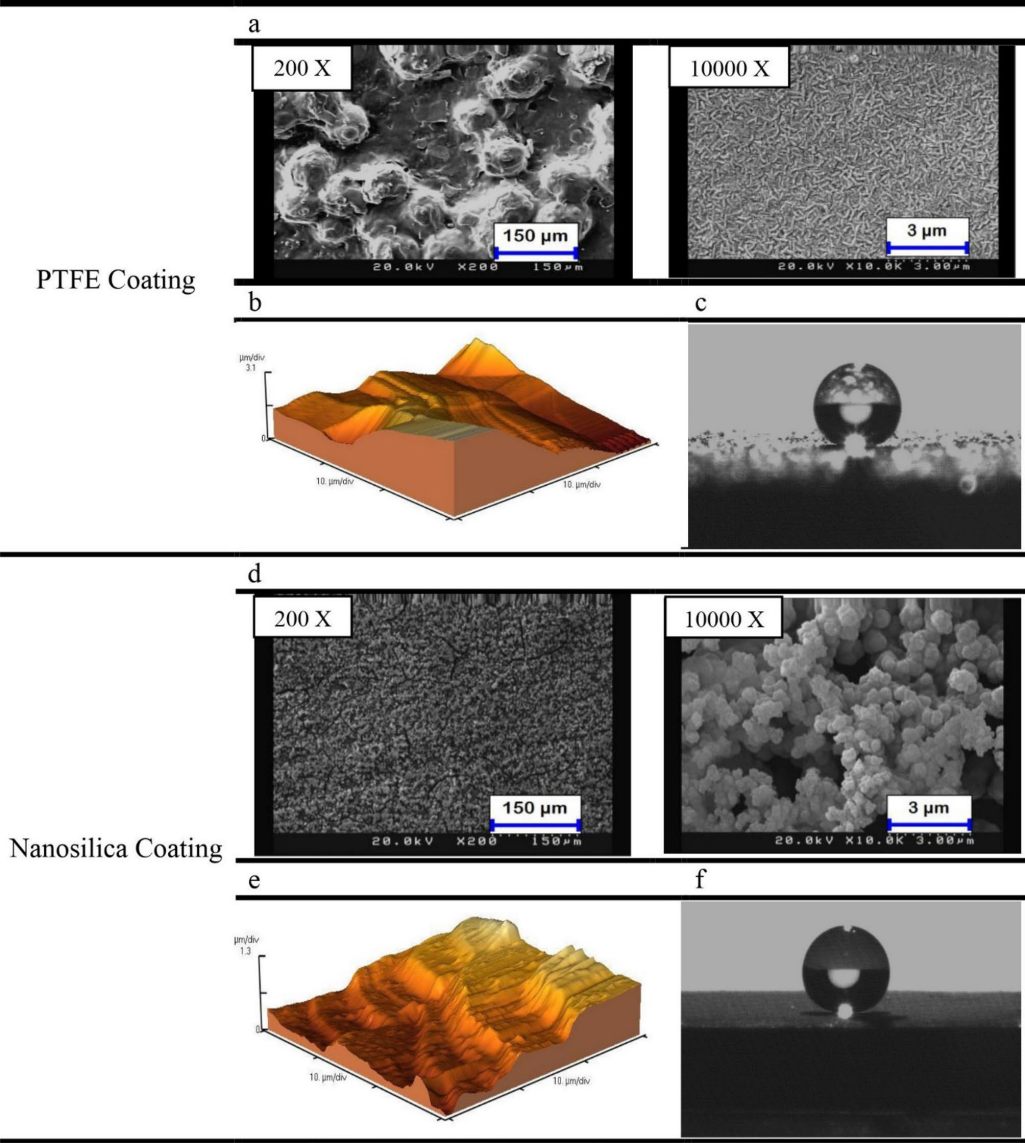
(**a**,**d**) SEM images and (**b**,**e**) images of 3D AFM (10 × 10 µm^2^) and (**c**,**f**) WCA images for PTFE and nanosilica coatings.

**Figure 2:**
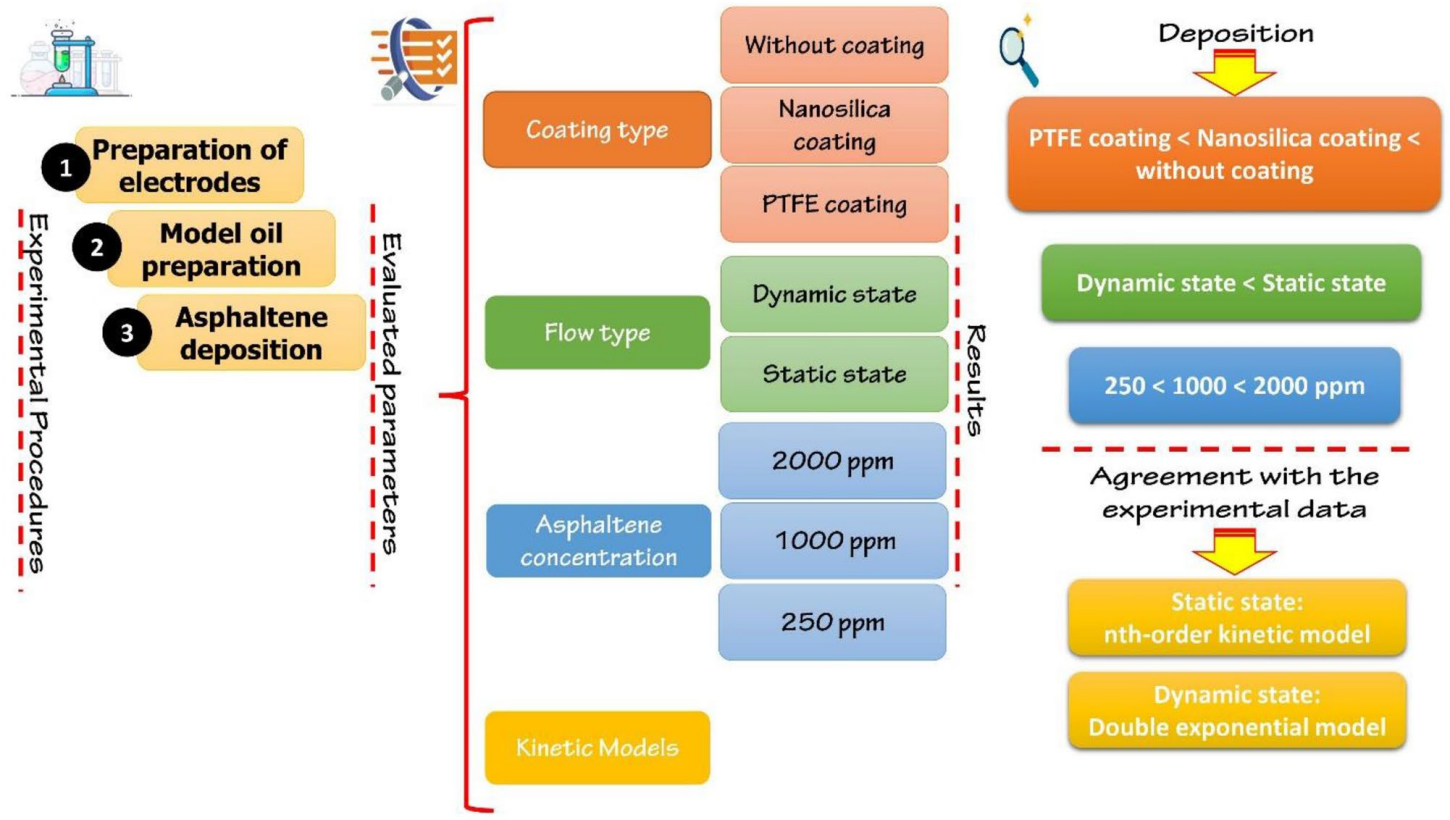
The procedure of experiments conducted in this study.

The electrodes used in this study are made of low-carbon steels with 0.15–0.30% carbon. Metal blades were cut from a sheet with a thickness of 0.1 cm in dimensions of 2.5 cm × 10 cm. In this study, two types of electrodes, including PTFE and nanosilica coated electrodes, were used to examine the asphaltene deposition on superhydrophobic surfaces. PTFE coating includes a primer and an overcoat layer. In order to produce superhydrophobic PTFE coating with hierarchical structure and low surface energy, glass beads microparticles with an average size of 82 microns were used in the primer layer. This coating was applied on the metal surfaces by spraying process at a pressure range of 50–100 Psi. For the second type of coating, the superhydrophobic nanosilica coating was applied on the metal surfaces by a one-step electrodeposition process of a sol–gel, which contained tetraethoxysilane (TEOS) (purity ≥ 98.5%) and dodecyltrimethoxysilane (DTMS) (purity > 93%) as mixed sol–gel precursors. The precursor solution contains 1 ml TEOS, 1 ml DTMS, 10 ml potassium nitrate, and 40 ml ethanol. The solution pH was adjusted to 4 by hydrochloric acid. This solution was pre-hydrolyzed for 12 h at room temperature via strong stirring. The electrodeposition process was then performed on the surface of the metal blades. The wetting properties of the coated and uncoated surfaces are obtained by measuring the contact angle (CA) and sliding angle (SA). The CA was measured on several different samples of uncoated surfaces. The uncoated surfaces had CA of 57°–97° and SA more than 90°, and therefore had neutral and somewhat hydrophilic wetting properties (the CA reported in this study is the average of five measurements at different points on the coated and uncoated surfaces). This suggests that asphaltene deposition tests have been performed on uncoated surfaces with neutral to hydrophilic wetting properties. While both PTFE coating and nanosilica coating produced superhydrophobic surfaces, the PTFE coating reached a CA of 152 ± 0.220° and a SA of 3 ± 0.376° and a CA of 166 ± 0.481° and SA of nearly zero were obtained for nanosilica coating. It is worth noting that, what is important for the reproduction of PTFE coating processes in the spray method is the correct selection of micro particle size distribution, control of the nozzle distance from the surface and the injection pressure. In the reproduction of nanosilica coating by electrodiposition method, precise regulation of current density and precise control of sol–gel solution pH are important. In our study, many samples were made by each method and for all of them the wettability properties were measured and compared and in all cases the results were very consistent. The surface roughness plays a major role in wettability. An atomic force microscope (AFM) (CP II, Veeco) was used to measure the roughness of two representative coated surfaces. The scan range was 10 × 10 µm^2^. Figure 1 shows surface morphology images (Fig. 1a,d), 3D AFM images (Fig. 1b,e), and water contact angle (WCA) images **(**Fig. 1c,f) of PTFE and nanosilica coatings. The procedure of the current study is shown in Fig. 2.

### Model oil preparation

Toluene and normal heptane were purchased with a purity of 99%. Crude oil samples were obtained from one of the oil reservoirs located in the southwest of Iran. The asphaltene was extracted from a crude oil sample according to the IP-143 standard method. Additional information on the composition of asphaltene extracted from crude oil based on H/C ratio, FTIR aromatic index, the heteroatoms content, the number of aliphatic chains and the XRD aromatic index are available in the literature^31^. The dried asphaltene powder was first dissolved in toluene by sonication at 40 kHz and then normal heptane was gradually added to the solution on a magnetic stirrer. The mixture was sonicated again for 5 min and was kept static for 24 h to achieve equilibrium. In this study, three different levels of asphaltene concentrations including 2000 ppm, 1000 ppm, and 250 ppm with toluene/n-heptan ratio of 1.6/38.4 (4% toluene concentration) were used to investigate the electrodeposition of asphaltene on coated and uncoated surfaces. These concentration values are initial concentrations. These values have been selected based on previous studies^32^. It should be noted that this mixture was prepared at ambient pressure and temperature. Although asphaltene particles in real crude oil show a neutral charge^33–35^, their charge in heptol solution depends on the concentration of toluene in the solution. It is worth mentioning that the electrodeposition process here was only used to simulate unstable asphaltene deposition.

### Asphaltene deposition process on surfaces of the electrodes

**Figure 3:**
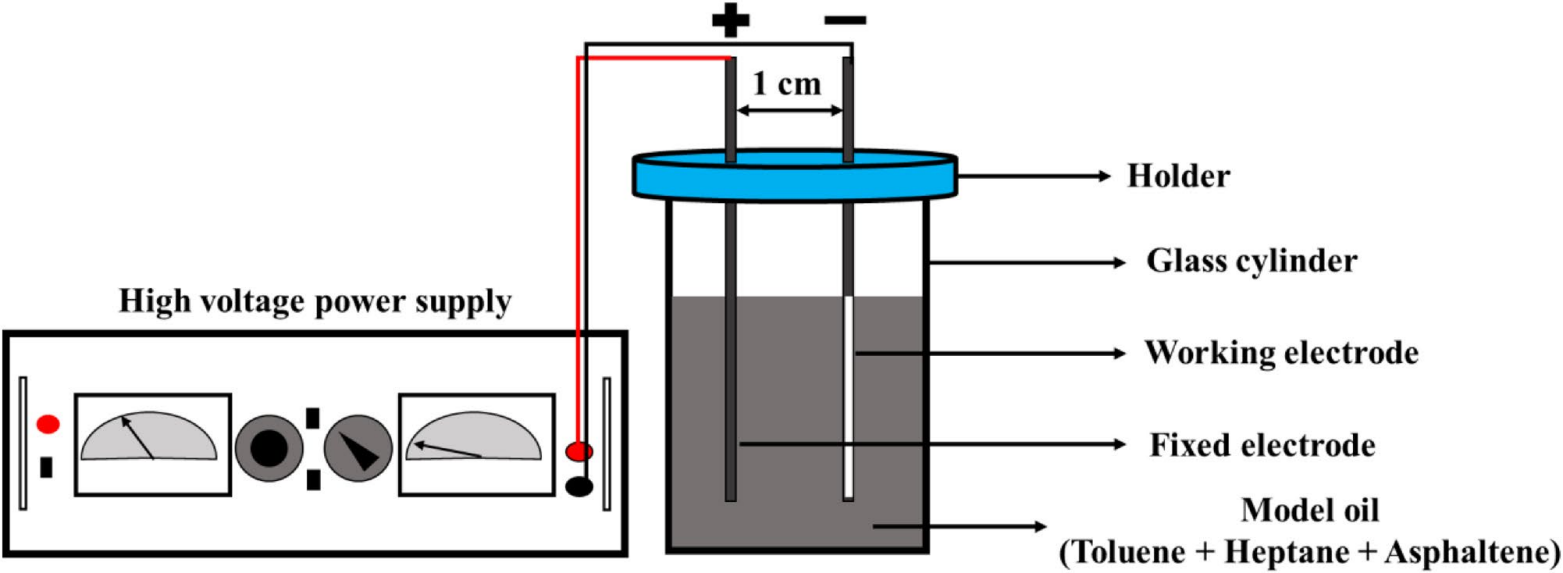
A schematic of the electrodeposition system.

Given the mycelial structure of asphaltenes and the presence of heteroatoms and metal elements such as nickel and vanadium in the constituent structure of asphaltene molecules and functional groups, it can be assumed that these compounds have a charge^36^. Numerous experiments have shown that asphaltenes in the electric field are directly affected by the force of the electric field^37,38^. In one comprehensive study, conducted by Hosseini et al.^39^, the effect of electric fields with different strengths on three different asphaltene samples was investigated. The main purpose of this study was to determine the amount of aggregation rate and aggregation size of asphaltene particles in the electrostatic field which was done using the visual inspection method. Based on the results obtained, the higher the aggregation rate the aggregation size of asphaltene particles in the electrostatic field may cause faster deposition. According to studies conducted in the literature, in this study, an electrical deposition cell was used to simulate the asphaltene deposition on coated and uncoated surfaces. The electrical deposition cell used in this study contains 40 ml of solution in a static state, and in dynamic state experiments, a shear rate was applied using a stirring magnet in 50 ml of solution. Figure 3 shows a schematic of the device used to perform the asphaltene deposition process on the coated and uncoated electrodes. Two metal electrodes are held in parallel by a removable plastic cap. A high voltage power supply device (Oltronix LS 529R) was used to convert alternating current (AC) to direct current (DC) and create an electric field between two electrodes. During the asphaltene deposition process, an uncoated blade, which is fixed in all experiments, plays the role of the anode, and the coated and uncoated blades play the role of the cathode. In order to measure the amount of asphaltene deposition, all blades were numbered first and the weight of each was measured and recorded using the analytical weighing scale with ± 0.0001 g accuracy. The weight of the electrodes used in this study was 30 g on average. At the end of each test, after removing the blades from the solution, they were placed at room temperature to be dried completely. After drying, a uniform blackish-brown layer of asphaltene deposit was being observed on the surface of the blade. The weight of these blades was measured and recorded at the end of the experiment and after drying at room temperature. The difference between the weight of the blades at the end of the experiment (blades containing asphaltene deposition) and their weight at the beginning of the experiment (clean blades) indicates the amount of asphaltene deposition. The strength of the electric field was determined based on experiments performed on the uncoated sample. Based on the obtained results, the amount of asphaltene deposition on uncoated electrodes at an electric field strength of 2 kV/cm reached its maximum^32^. For this reason, in this study, 2 kV/cm electric field strength was used to maximize the amount of deposition. In order to investigate the asphaltene deposition on coated and uncoated surfaces at different concentrations and different flow states, each electrode was exposed to an electric field at 5, 10, 20, 40, 60, 80, 120, 180 and 300 s, and the amount of asphaltene deposition at any time and in any situation was assessed. It should be noted that in order to measure the asphaltene deposition at any time, a new experiment was conducted. The experiments were repeated three times for each deposition condition, and the average values of these runs are reported here.

## Kinetic models

In general, measuring, predicting, and understanding deposition rate in engineering sciences is very important. Some efforts^40–43^ have been made to model the asphaltene deposition based on deposition kinetic for selecting the optimal operating conditions and the treatment of asphaltene deposition at the field scale. All these studies were conducted for uncoated surfaces^44,45^.

In order to describe the results of static and dynamic experiments at different concentrations and times, the double exponential model, diffusion equation model, Elovich’s equation model, nth-order kinetics and modified second-order models were used. Although these models are suitable for modeling the adsorption process, the application of these models in this study could also provide a good insight into the kinetics of the adsorption/deposition process.

### Double exponential model

This model was developed in 1993 by Wilczak and Keinath^46^ and is used to describe the adsorption process with respect to both chemical and mathematical perspectives. The model links the two-step mechanism of the fast and slow adsorption process^47^ as given in Eq. (1):

1 $${Q}_{t}={Q}_{e}-\frac{{D}_{1}}{{m}_{ads}}exp\left(-{K}_{D1}t\right)-\frac{{D}_{2}}{{m}_{ads}}exp\left(-{K}_{D2}t\right),$$

where *Q*_*t*_ and *Q*_*e*_, are the amount of asphaltenes at each time point of contact and the amount of asphaltene adsorbed on the surface of the electrode at equilibrium (mg/cm^2^), respectively. *D*1 and *D*2 are asphaltene fast and slow adsorption fraction (mg/l), *t* is time, *K*_*D*1_ and *K*_*D*2_ are fast and slow rate constants (min^−1^) and *K*_*D*1_ is larger than *K*_*D*2_. It is worth noting that the sum of the two parameters *D*_1_/*m*_*ads*_ and *D*_2_/*m*_*ads*_ is the physical equivalent of the calculated value of *Q*_*e*_. The rate of absorption of the absorbing material in both slow and fast states is expressed by *SF* and *RF*, respectively, and is expressed by Eqs. (2) and (3).

2 $$SF=100\left[\frac{{D}_{2}}{\left({D}_{1}+{D}_{2}\right)}\right].$$

3 $$RF=100\left[\frac{{D}_{1}}{\left({D}_{1}+{D}_{2}\right)}\right].$$

### Diffusion equation model

The penetration of adsorbed molecules or ions into the pores is considered in order to find the appropriate kinetic model for the porous adsorbents. In many cases, the rate of absorption of a sorbent is controlled by the amount of penetration into the particles^48^. Equation (4) was expressed by Weber and Morris for this purpose.

4 $${Q}_{t}={k}_{p}{t}^\frac{1}{2}+I.$$

In this equation, *k*_*p*_ is defined as the diffusion rate coefficient and its unit is [mg/(cm^2^ × min^0.5^)]. This rate coefficient could be obtained from the slope of the plots (*Q*_*t*_ vs. *t*^0.5^), and *I* is the intercept.

### Elovich’s equation model

This equation was introduced by Zeldowitsch in 1934 for absorption based on chemical bonding mechanism^49^. This equation is expressed by Eqs. (5) and (6).

5 $${Q}_{t}=\left(\frac{2.3}{a}\right)\mathrm{log}\left(t+{t}_{0}\right)-\left(\frac{2.3}{a}\right)\mathrm{log}\left({t}_{0}\right),$$

6 $${t}_{0}=\frac{1}{\alpha a}.$$

In this equation, *Q*_*t*_ is the amount of asphaltene adsorbed at time *t*, *α* is the initial adsorption value in gram and *a* is adsorption constant.

### nth-Order kinetics model

In general, direct calculation of the rate constant and order of the adsorption reaction is more appropriate than assuming the reaction order, *n*, as 1 or 2, and therefore, using the nth-order kinetic model is much more efficient^50^. This model is expressed by Eq. (7).

7 $${Q}_{t}={Q}_{e}\left\{1-{\left[\frac{1}{{\beta }_{n}+{k}_{n}\left(n-1\right)t}\right]}^{\frac{1}{\left(n-1\right)}}\right\},$$

where *k*_*n*_ is the rate constant and its unit depends on the reaction order (1/min)(mg/cm^2^)^1−n^, *β*_*n*_ is the impurity, pre-adsorbed on the surface and is defined by Eq. (8).

8 $$\beta _{n} = {\raise0.7ex\hbox{$1$} \!\mathord{\left/ {\vphantom {1 {\left( {1 - \theta _{0} } \right)^{{n - 1}} }}}\right.\kern-\nulldelimiterspace} \!\lower0.7ex\hbox{${\left( {1 - \theta _{0} } \right)^{{n - 1}} }$}},$$

where *θ*_0_ is a dimensionless surface coverage in the pre-adsorption step and is expressed by (*θ*_0_ = *Q*_*0*_*/Q*_*e*_).

### Modified second-order model

Using the nth-order kinetic equation for *n* = 2, a modified second-order equation can be obtained^50^. This model is defined by Eq. (9).

9 $${Q}_{t}={Q}_{e}\left[1-\frac{1}{\beta +kt}\right].$$

## Results and discussion

In this section, the effect of two different coatings and asphaltene concentration on the amount of asphaltene deposition at different flow states are analyzed and the results are discussed. Finally, we investigate the effect of these factors on the kinetics of asphaltene deposition on electrode surfaces. Table 1 summarizes the most important results obtained in each section.

**Table 1:** Summary of the most important results.

Evaluated parameter	Constant values	Results	
Coating type	Ambient pressure and temperature, Asphaltene concentration 2000 ppm, Electric field strength 2 kV/cm, Static state	Asphaltene deposition	PTFE coating ˂ Nanosilica coating ˂ Without coating electrode
Type of flow state	Ambient pressure and temperature, Asphaltene concentration 2000 ppm, Electric field strength 2 kV/cm, End of 300 s of exposure to electric filed		For without coating electrode and PTFE coating: Dynamic state ˂ Static state
Asphaltene concentration	Ambient pressure and temperature, Electric field strength 2 kV/cm, PTFE coating		At 2000, 1000 and 250 ppm: Dynamic state ˂ Static state
Coating type + Asphaltene concentration	Ambient pressure and temperature, Electric field strength 2 kV/cm, End of 300 s of exposure to electric filed, Static state		PTFE coating-250 ppm ˂ PTFE coating-1000 ppm ˂ PTFE coating-2000 ppm ˂ Nanosilica coating-2000 ppm ˂ Without coating-2000 ppm
	Ambient pressure and temperature, Electric field strength 2 kV/cm, End of 300 s of exposure to electric filed, Dynamic state		PTFE coating-250 ppm ˂ PTFE coating-1000 ppm ˂ PTFE coating-2000 ppm ˂ Without coating-2000 ppm
Kinetics models	Ambient pressure and temperature, Electric field strength 2 kV/cm	Calculated Q_e_ ≈ experimental Q_e_ Maximum amount of deposition: During the first 2 min Deposition reduction ≈ 56% (static state, 2000 ppm) Deposition reduction ≈ 70% (dynamic state, 2000 ppm) Best agreement with the experimental data: nth-order kinetics model in static state, double exponential model in dynamic state	

### The effect of coating type on asphaltene deposition in the static state

**Figure 4:**
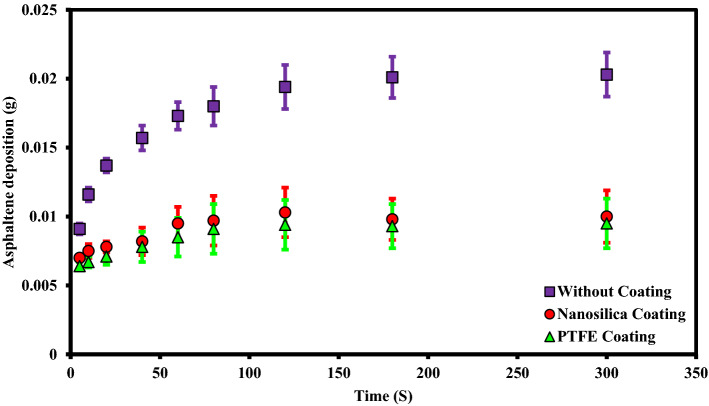
Asphaltene deposition rate on uncoated and coated electrodes for different exposure times to electric field at ambient pressure and temperature (Asphaltene concentration 2000 ppm, Electric field strength 2 kV/cm).

These experiments were performed at static state and asphaltene concentration of 2000 ppm for uncoated, PTFE coated and nanosilica coated electrodes. During these experiments, other influential parameters such as time, toluene concentration, asphaltene concentration, type of flow state, and voltage were kept constant. The parameters used in the design of the experiment are listed in Table 2. The rate of asphaltene deposition on three types of electrodes as a function of exposure time to the electric field with 2 kV/cm strength, is shown in Fig. 4. It is necessary to mention that, each data point on the asphaltene deposition rate curve, as shown in Fig. 4, was generated from an independent test. The amounts of asphaltene deposition, in this case, are shown in Table 3.

**Table 2:** The design of experiments to investigate the effect of coating type on asphaltene deposition in static sate.

		Test number	Type of working electrode
Parameter	Parameter	1	Without coating
		2	PTFE coating
		3	Nanosilica coating
	Constant	Electric field application time (5, 10, 20, 40, 60, 80, 120, 180, 300 s) Electric field strength 2 kV/cm, 4% toluene concentration, 2000 ppm asphaltene concentration, static state

**Table 3:** Asphaltene deposition on uncoated, PTFE-coated and nanosilica coated electrodes for different exposure times to electric field.

Test number	Test duration (s)	Without coating		PTFE coating		Nanosilica coating	
		Weight (g)	Percent	Weight (g)	Percent	Weight (g)	Percent
1	5	0.0091	11.3	0.0064	8.0	0.0070	8.7
2	10	0.0116	14.5	0.0067	8.3	0.0075	9.3
3	20	0.0137	17.1	0.0071	8.8	0.0078	9.7
4	40	0.0157	19.6	0.0078	9.7	0.0082	10.2
5	60	0.0173	21.6	0.0085	10.6	0.0095	11.8
6	80	0.0180	22.5	0.0091	11.3	0.0097	12.1
7	120	0.0194	24.2	0.0094	11.7	0.0103	12.8
8	180	0.0201	25.1	0.0093	11.6	0.0098	12.2
9	300	0.0203	25.3	0.0095	11.8	0.0100	12.5

Deposition value (electric field strength 2 kV/cm, 4% toluene concentration, 2000 ppm asphaltene concentration, static state).

Based on our observations, the amount of asphaltene deposition on the surface of coated electrodes is lower than that of the uncoated electrode. Although the difference is not significant, the amount of asphaltene deposition on the surface of the electrode with PTFE coating is lower than that of nanosilica. It was also observed that the asphaltene deposition on the coated surfaces has low adhesion and could be easily removed from the surface after taking it out of the oil sample solution. It is worth recalling that, the asphaltene is the most polar component of crude oil and contains large amounts of active species^51^. They are known as key components of surface wettability change through the interaction of its polar functional groups with polar sites on a solid surface^52,53^. For coating created by the electrodeposition process, the wettability of the coatings largely depends on several parameters such as electrodeposition conditions of the coating such as charge transferred, applied voltage, , the alloy type and roughness of the working electrode and the surface energy of the coating^54^. Lower or negative surface energy values associated with lower or negative adhesion tendencies would be a more effective system for reducing asphaltene deposition^55^. The use of some polymer-based coatings with suitable chemically inert properties could reduce the tendency for severe asphaltene adhesion^55^. The coatings used to prevent or reduce asphaltene deposits must have the required surface characteristics for this purpose. The creation of superhydrophobic coatings with low surface energy could change the solid surface sites to non-polar^56,57^ and ultimately reduces the tendency of asphaltene deposition on the coated surfaces^58–64^. Therefore, such a surface can not only prevent the deposition of minerals in formation water, but also can significantly reduce the deposition of asphaltene. Superhydrophilic surfaces could also effectively block the access of asphaltene to the surface by creating a water film, and therefore reduce the asphaltene deposition^65^, however, having a water film on the surface could initiate the deposition of inorganic scales and creation of suitable sites for organic deposits and also corrosion.

### The effect of flow type on the amount of asphaltene deposition

**Figure 5:**
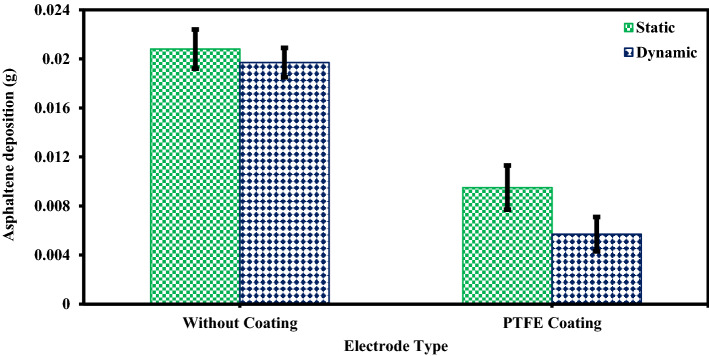
Comparison of asphaltene deposition on uncoated and PTFE coated electrodes after 300 s of exposure to electric filed and for asphaltene concentration of 2000 ppm at ambient pressure and temperature.

An experiment was designed to investigate the effect of shear rate on the amount of asphaltene deposition on electrode surfaces with and without superhydrophobic coating. In this regard, two parameters (flow state and type of working electrode) were considered as variables. Experiments were performed for two types of flow and working electrodes (without coating electrode and PTFE superhydrophobic coating electrode). In a dynamic state, the oil sample solution was agitated using a magnetic stirrer at 400 rpm and the amount of deposition on the surfaces with and without coating was measured. During these experiments, other influential parameters such as voltage, toluene concentration, asphaltene concentration, and time were kept constant. The parameters adjusted in the design of the experiment are shown in Table 4. The amount of asphaltene deposition on uncoated and superhydrophobic PTFE coated electrodes under static and dynamic conditions after 300 s of electrodeposition is shown in Fig. 5. The detailed test results are reported in Table 5. It should be noted that measurements were made at ambient pressure and temperature. According to the results, hydrophobic properties decrease the adhesion force between the surface and the deposition. The amount of asphaltene deposition in the dynamic state for the uncoated and the superhydrophobic PTFE-coated electrodes is far less than in other cases. Dynamic deposition for PTFE superhydrophobic coating is far less than the other cases.

**Table 4:** The design of experiments to investigate the effect of flow type on the amount of asphaltene deposition.

		Test number	Flow state	Type of working electrode
Parameter	Variable	1	Static	Without coating
				PTFE coating
		2	Dynamic	Without coating
				PTFE coating
	Constant	Electric field application time (5, 10, 20, 40, 60, 80, 120, 180, 300 s) Electric field strength 2 kV/cm, 4% toluene concentration, 2000 ppm asphaltene concentration

**Table 5:** Asphaltene deposition rate for uncoated and PTFE coated electrodes in static and dynamic states.

Test number	Test duration (s)	Without coating				PTFE coating			
		Static		Dynamic		Static		Dynamic	
		Weight (g)	Percent	Weight (g)	Percent	Weight (g)	Percent	Weight (g)	Percent
1	5	0.0091	11.3	0.0078	9.7	0.0064	8.0	0.0051	6.3
2	10	0.0116	14.5	0.0096	12.0	0.0067	8.3	0.0058	7.2
3	20	0.0137	17.1	0.0102	12.7	0.0071	8.8	0.0063	8.8
4	40	0.0157	19.6	0.0142	17.7	0.0078	9.7	0.0069	8.6
5	60	0.0173	21.6	0.0151	18.8	0.0085	10.6	0.0077	9.6
6	80	0.0180	22.5	0.0169	21.1	0.0091	11.3	0.0084	10.5
7	120	0.0194	24.2	0.0183	22.8	0.0094	11.7	0.0086	10.7
8	180	0.0201	25.1	0.0191	23.8	0.0093	11.6	0.0088	11.0
9	300	0.0203	25.3	0.0190	23.7	0.0095	11.8	0.0087	10.8

Deposition value (electric field strength 2 kV/cm, 4% toluene concentration, 2000 ppm asphaltene concentration).

### The effect of asphaltene concentration on the amount of asphaltene deposition

**Figure 6:**
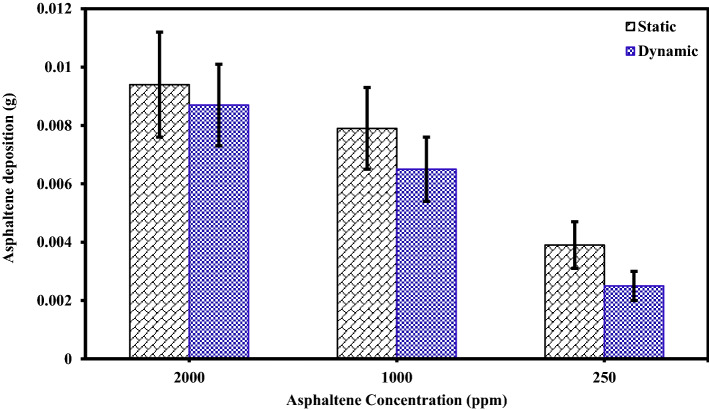
The effect of asphaltene concentration on asphaltene deposition for PTFE coating in static and dynamic states at ambient pressure and temperature.

**Figure 7:**
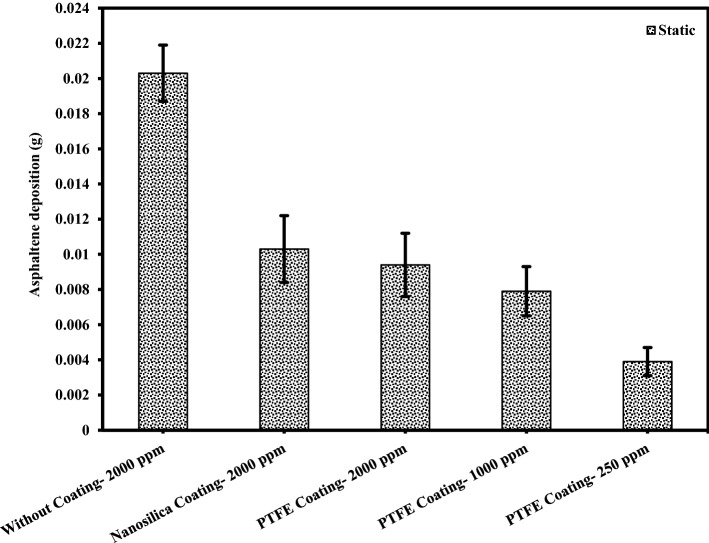
Comparison of coated and uncoated electrodes based on the amount of deposition at different asphaltene concentrations in static state at ambient pressure and temperature (The amount of asphaltene deposition were reported at the end of 300 s period).

**Figure 8:**
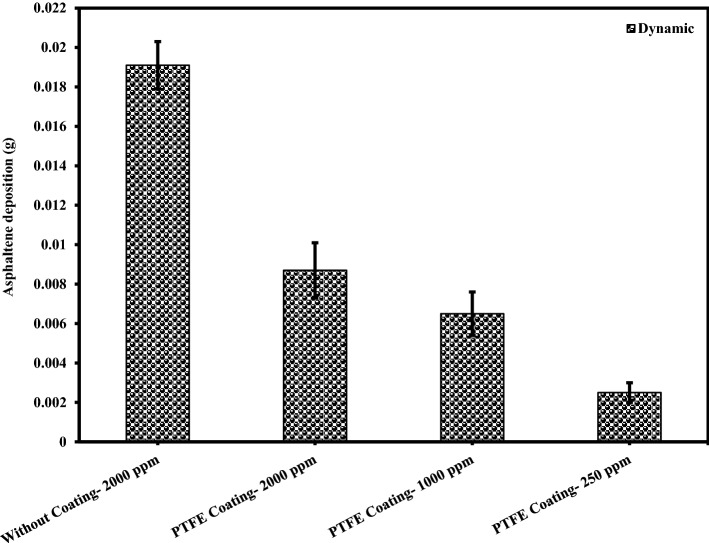
Comparison of coated and uncoated electrodes based on the amount of deposition at different asphaltene concentrations in dynamic state at ambient pressure and temperature (The amount of asphaltene deposition were reported at the end of 300 s period).

An experiment was designed to investigate the kinetics of asphaltene deposition on superhydrophobic PTFE-coated electrodes for three different asphaltene concentrations. During these experiments, other influential parameters such as voltage, toluene concentration and coating type were kept constant. The parameters used in the design of the experiment are listed in Table 6. Experiments were performed for asphaltene concentrations of 2000, 1000 and 250 ppm and different exposure times up to 5 min. The amount of asphaltene deposition as a function of asphaltene concentration in the static and dynamic states for the PTFE superhydrophobic coated electrodes is shown in Fig. 6. The detailed information for this experiment can be found in Tables 7 and 8. Figures 7 and 8 also compare the amount of deposition at different asphaltene concentrations in static and dynamic states. It is observed that decreasing the asphaltene concentration and the duration of the electrodeposition process decrease the amount of asphaltene deposition in the static and dynamic states for PTFE superhydrophobic coatings. The amount of asphaltene deposition at all three concentrations and dynamic state is lower than that in the static state. As shown in Fig. 7, the amount of asphaltene deposition after 300 s at a concentration of 2000 ppm for PTFE superhydrophobic coating is lower than that for nanosilica coating. The reason for this result can be related to the functional groups on the surface of nanosilica coatings and asphaltene. H-bonding sites of surface hydroxyls, in the nanosilica superhydrophobic coating, formed by modification with silane material (DTMS), can be effective in adsorbing active groups in the asphaltene surface such as carboxylic. Some studies have also confirmed the effect of interaction between surface active sites of asphaltenes and sorbent surface active sites^61^. In PTFE coating, there is no interaction between fluorine in PTFE coating and asphaltene particles, and therefore the amount of asphaltene deposition in PTFE superhydrophobic coating will be less than nanosilica superhydrophobic coating. In this experiment, due to high the concentration and opacity of the solution, the movement of asphaltene particles at a concentration of 2000 ppm at the beginning and end of the experiment was not observed. As the concentration of asphaltene decreased, the movement of the particles in the form of dark masses toward the electrodes became evident. In this experiment, the amount of asphaltene deposition in a short time on the superhydrophobic coated electrodes, especially PTFE superhydrophobic coated electrode, at the static state and 250 ppm, was very low and close to zero, and no traces of deposition were observed in a short time (Table 7). In contrast, the deposition rate at 250 ppm, in the static state on the uncoated electrode, was significantly high (Supplementary Fig. S.1). Experiments with dynamic states showed very small deposition rates at longer times for superhydrophobic PTFE coating (Table 8 and Fig. 8). It can be inferred that the hydrophobic property of the coating reduces the crude oil affinity for sticking to the surface^26^ and therefore, at lower concentrations the amount of asphaltene deposition is very low. It should be noted that the reason for a high CA and low SA in superhydrophobic surfaces is the low surface energy along with the hierarchical structure of the surface. As these two properties are enhanced at the surface, the existing surface becomes more hydrophobic until it reaches the superhydrophobicity^66^. The superhydrophobic and low sliding angle characteristic of the produced PTFE superhydrophobic coating could be considered as the main reason for reducing asphaltene deposition. The surface roughness of the coatings was measured using the AFM method, and some roughness characteristics including average surface roughness (Ave Rough), root mean square roughness (RMS Rough) and mean height roughness (Mean Ht) of the samples were calculated. Figure 1b,e show 3D roughness images of PTFE and nanosilica superhydrophobic coatings measured by AFM. The roughness characteristics of the coatings are listed in Supplementary Table S.1. As can be seen in this table, the average surface roughness of PTFE and nanosilica superhydrophobic coatings is 1.255 µm and 611.2 nm, respectively. The roughness plays a major role in surface wettability and consequently in asphaltene deposition. Our objective in synthesizing the coatings was to obtain superhydrophobic surfaces with very low sliding angles. It was achieved by combining low surface energy and desired roughness in PTFE superhydrophobic coatings and generating a rough surface and later modifying the surface energy in nanosilica coating. Creating roughness on the surface increases the surface area and then reduces its energy. Therefore, low surface energy is considered as the main factor in superhydrophobic properties of the surface and roughness is the aggravating factor^67^.

**Table 6:** The design of experiments to investigate the effect of concentration on the amount of asphaltene deposition.

		Test number	Asphaltene concentration (ppm)	Asphaltene content (g)	Flow state
Parameter	Variable	1	2000	0.08	Static
					Dynamic
		2	1000	0.04	Static
					Dynamic
		3	250	0.01	Static
					Dynamic
	Constant	Electric field application time (5, 10, 20, 40, 60, 80, 120, 180, 300 s) Electric field strength 2 kV/cm, 4% toluene concentration, PTFE coating

**Table 7:** Asphaltene deposition rate on PTFE coated electrode in static state for different asphaltene concentrations and exposure time to electric filed.

Test numbe	Test duration (s)	Concentration (2000 ppm)		Concentration (1000 ppm)		Concentration (250 ppm)	
		Weight (g)	Percent	Weight (g)	Percent	Weight (g)	Percent
1	5	0.0064	8.0	0.0042	10.5	0.0014	14.0
2	10	0.0067	8.3	0.0044	11.0	0.0018	18.0
3	20	0.0071	8.8	0.0047	11.7	0.0022	22.0
4	40	0.0078	9.7	0.0052	13.0	0.0026	26.0
5	60	0.0085	10.6	0.0061	15.2	0.0031	31.0
6	80	0.0091	11.3	0.0062	15.5	0.0035	35.0
7	120	0.0094	11.7	0.0078	19.5	0.0039	39.0
8	180	0.0093	11.6	0.0080	20.0	0.0041	41.0
9	300	0.0095	11.8	0.0079	19.7	0.0038	38.0

Deposition value (electric field strength 2 kV/cm, 4% toluene concentration, PTFE coating, static state).

**Table 8:** Asphaltene deposition rate for PTFE coated electrode in dynamic state for different asphaltene concentrations and exposure time to electric field.

Test number	Test duration (s)	Concentration (2000 ppm)		Concentration (1000 ppm)		Concentration (250 ppm)	
		Weight (g)	Percent	Weight (g)	Percent	Weight (g)	Percent
1	5	0.0051	6.3	0.0033	8.2	0.0006	6.0
2	10	0.0058	7.2	0.0036	9.0	0.0007	7.0
3	20	0.0063	7.8	0.0039	9.7	0.0013	13.0
4	40	0.0069	8.6	0.0044	11.0	0.0016	16.0
5	60	0.0077	9.6	0.0053	13.2	0.0018	18.0
6	80	0.0084	10.5	0.0057	14.2	0.0023	23.0
7	120	0.0086	10.7	0.0062	15.5	0.0025	25.0
8	180	0.0088	11.0	0.0072	18.0	0.0028	28.0
9	300	0.0087	10.8	0.0065	16.2	0.0024	24.0

Deposition value (electric field strength 2 kV/cm, 4% toluene concentration, PTFE coating, dynamic state).

### Modeling of asphaltene deposition kinetics on coated and uncoated electrodes

The kinetic behavior of asphaltene deposition was modeled based on the results obtained in the previous sections. These models are appropriate for adsorption processes, however, their usage for deposition/adsorption processes (this study), provides a suitable insight into the kinetics of the deposition/adsorption process. Table 9 shows the parameters of the kinetic models for the coated and uncoated electrodes at different concentrations. The results of the deposition model are as follows.

**Table 9:** Parameters calculated for a variety of kinetic models.

Flow type	Static					Dynamic			
C_i_ (initial conc.), ppm	2000 ppm			1000 ppm	250 ppm	2000 ppm		1000 ppm	250 ppm
Type of coating	Without coating	Nano silica	PTFE coating	PTFE coating	PTFE coating	Without coating	PTFE coating	PTFE coating	PTFE coating
Q_e_, exp. (mg/g)	0.0203	0.0100	0.0095	0.0079	0.0038	0.0190	0.0087	0.0065	0.0024
**Modified second-order model**									
Q_e_, (mg/g)	0.0223	0.0103	0.0106	0.0098	0.0045	0.0209	0.0092	0.0084	0.0023
k_2_ (min^−1^)	0.0633	0.0616	0.0572	0.0153	0.0450	0.0376	0.0502	0.0205	0.0438
β	1.4346	2.6511	2.4077	1.7292	1.2896	1.3210	1.8605	1.5646	1.0834
RMSE	0.0022	0.0020	0.0020	0.0013	0.0004	0.0017	0.0014	0.0010	0.0003
AAPRE	3.7993	4.0539	5.1657	5.6452	8.1876	5.0662	4.2933	5.3239	13.3185
**Double exponential model**									
Q_e_, (mg/g)	0.0219	0.0118	0.0093	0.0105	0.0052	0.0194	0.0087	0.0070	0.0038
D_1_	0.0101	0.0014	0.0030	0.0014	0.0019	0.0128	0.0041	0.0025	0.0015
D_2_	0.0032	0.0027	0.0008	0.0047	0.0021	0.0000	0.0000	0.0014	0.0018
K_D1_	0.0334	0.0304	0.0269	0.0103	0.0391	0.0204	0.0261	0.0170	0.0247
K_D2_	0.0039	0.0039	0.0039	0.0043	0.0032	0.0043	0.0039	0.0083	0.0036
RF, %	76.0460	35.4133	78.8023	23.7974	46.9985	100.0000	100.0000	63.7058	44.4081
SF, %	23.9539	64.5866	21.1976	76.2025	53.0014	0.0000	0.0000	36.2941	55.5918
RMSE	0.0028	0.0024	0.0017	0.0014	0.0004	0.0021	0.0014	0.0009	0.0002
AAPRE	4.0802	6.6685	5.4571	5.3771	8.2962	2.4953	1.7973	3.0413	7.9853
Q_e_, exp. (mg/g)	0.0203	0.0100	0.0095	0.0079	0.0038	0.0190	0.0087	0.0065	0.0024
**nth-order kinetic model**									
Q_e_, cal., (mg/g)	0.0228	0.0100	0.0095	0.0084	0.0041	0.0239	0.0086	0.0072	0.0025
β_n_	1.5796	1.0163	1.0541	1.1881	1.0593	1.5306	1.0854	1.0993	1.0346
k_n_ (min^−1^)	0.0794	0.0295	0.0210	0.0114	0.0251	0.0422	0.0251	0.0166	0.0467
n	2.5434	1.0153	1.0525	1.1920	1.2680	2.4707	1.1091	1.1693	1.5429
RMSE	0.0018	0.0020	0.0019	0.0013	0.0003	0.0021	0.0014	0.0010	0.0002
AAPRE	1.3565	2.3117	1.2594	3.3033	3.7448	5.4198	3.3659	5.0794	11.9815
**Elovich’s equation**									
Q_e_, (mg/g)	0.0225	0.0107	0.0106	0.0080	0.0045	0.0197	0.0095	0.0062	0.0025
a	1580.9478	873,019.4700	93,806.7807	16,475.2481	2291.0482	537.1581	32,803.0080	5943.5996	407.6974
α	323.0661	1147.4729	962.7946	1047.7282	1372.5937	316.1196	963.1319	1174.5895	1704.0341
RMSE	0.0008	0.0004	0.0006	0.0005	0.0003	0.0009	0.0003	0.0005	0.0003
AAPRE	4.7954	4.6750	6.7033	8.5840	11.0078	6.4137	4.7708	8.9519	15.5949
**Diffusion**									
Q_e_, (mg/g)	0.0240	0.0117	0.0125	0.0098	0.0059	0.0211	0.0112	0.0088	0.0044
K_p_	0.0008	0.0002	0.0003	0.0003	0.0002	0.0008	0.0003	0.0003	0.0002
I	0.0084	0.0067	0.0056	0.0034	0.0009	0.0066	0.0048	0.0025	0.0001
RMSE	0.0031	0.0022	0.0021	0.0012	0.0007	0.0027	0.0017	0.0011	0.0006
AAPRE	9.0855	6.1164	8.3095	7.6125	12.7893	10.0604	7.4261	7.6431	20.4254

#### Modified second-order model

In this model, *K*_2_ values for the static state were found to be 0.0153–0.0633 cm^2^/mg × min and 0.0205–0.0502 cm^2^/mg × min for the dynamic state. The values of *β*_2_ for the static state are in the range of 1.2896–2.6511 and for the dynamic state are in the range of 1.0834–1.8605.

#### Double exponential model

There are usually two steps in the asphaltene deposition process. The first involves the rapid deposition rate and the second step is related to the slow adsorption until equilibrium is reached. As the initial concentration of asphaltenes increases, this period will be longer. The initial rate of asphaltene build-up on a surface could be different from that at the later stages and this makes the two-step models a suitable choice for adsorption modeling. This behavior was also modeled in a study by Refs.^12,68^ who used quartz crystal microbalance with dissipation (QCM-D) measurements and examined the process of asphaltene adsorption for short and long times. Our results show that the *RF* value in static and dynamic states for the uncoated electrode is higher than that of the *SF*, indicating that this process is faster for the uncoated electrode. For the PTFE coating, the *RF* and *SF* values in static and dynamic states do not show any particular trend. In the dynamic state for the uncoated electrode at a concentration of 2000 ppm, the *RF* value is almost 100.0000 and the *SF* value is 0.0000 and in this state, the *RF* value for the PTFE coated electrode in 2000 ppm, is 100.0000 and the value of *SF* is 0.0000. This indicates that, under these conditions, the asphaltene deposition process happens only in the first step, i.e., during the rapid step and there is no slow deposition step under these conditions. The *RF* value for nanosilica coating and the concentration of 2000 ppm in a static state is lower than the *SF* value. This indicates that the slow deposition process is faster for this coating. The *RF* value for the dynamic state and the PTFE coating increases with increasing initial concentration (250, 1000, and 2000), and no specific trend is observed for the static state. The range of *K*_*D*1_ values for the static state and dynamic state is 0.0103–0.0391 and 0.0170–0.0261, respectively. Also, the range of *K*_*D*2_ values ​​for the static state is 0.0032–0.0039 and for the dynamic state is 0.0036–0.0083.

#### nth-Order kinetics model

The order of the deposition reaction, *n*, for the coatings in static and dynamic states decreases with the increasing initial concentration of asphaltene. Its value was calculated for the static state between 1.0153 and 1.2680 and for the dynamic state between 1.1091 and 1.5429. *K*_*n*_ values were calculated for static state in the range of 0.0114–0.0794 (1/min) × (mg/cm^2^)^1−n^ and 0.0166–0.0467 (1/min) × (mg/cm^2^)^1−n^ for the dynamic state. The *β*_*n*_ values for static and dynamic states were approximately 1. This means that initially there were no impurities or pre-adsorbed asphaltenes.

#### Elovich’s equation model

The deposition constant, *a*, for the static state and PTFE coatings is in the range 2291.0842–93,806.7807 and for the dynamic state and the PTFE coatings are in the range 407.6974–32,803.008 and increase with increasing concentration. Its value for nanosilica coating at static state and concentration of 2000 ppm is 873,019.4700 which is higher than 93,806.7807 for PTFE coating under these conditions. The value of this constant for the uncoated electrode at the static state and concentration of 2000 ppm is 1580.9478 and for this electrode at the dynamic state and concentration of 2000 ppm is 537.1581. Comparison of these results shows that by changing the flow state from static to dynamic, the deposition constant for the coated and uncoated electrodes decreases. Also, the initial deposition rate, *α*, for PTFE coatings at static and dynamic states increases with decreasing initial concentration. The value of this parameter for nanosilica coating in static state and concentration of 2000 ppm is 1147.4729; therefore, its value in nanosilica coating at constant state and concentration is higher than PTFE coating.

#### Diffusion equation model

*K*_*p*_ values for the uncoated electrode at 2000 ppm for static and dynamic states is 0.0008. The value of this parameter for PTFE coating in static and dynamic states at 1000 and 2000 ppm is 0.0003 and for 250 ppm is 0.0002. For nanosilica coating in a static state and 2000 ppm, its value is 0.0002, which is less than the corresponding value for PTFE coating. The values of *I* also decrease with decreasing initial concentration of asphaltenes for static and dynamic states and their values for PTFE coating in the static state are between 0.0009 and 0.0056 and for the dynamic state are between 0.0001 and 0.0048. The value for the nanosilica coating is 0.0067 and for the uncoated electrode at 2000 ppm, in a static state is 0.0084 and for the dynamic state is 0.0066. Comparison of the results for *K*_*p*_ and *I* show that their values in the dynamic state are always lower than those in the static state.

### Evaluation of kinetic models

In this study, root mean square error (*RMSE*) and average absolute percent relative error (*AAPRE*), as two statistical parameters, were used to evaluate and compare the accuracy of kinetic models. These parameters are calculated using Eqs. (10) and (11).

10 $$RMSE=\sqrt{\frac{1}{N}{\sum }_{i=1}^{N}{\left({d}_{exp,i}-{d}_{pred,i}\right)}^{2}},$$

11 $$AAPRE=\left(\frac{1}{N}{\sum }_{i=1}^{N}\frac{\left|{d}_{exp,i}-{d}_{pred,i}\right|}{{d}_{exp,i}}\right)\times 100.$$

Here, *d*_*exp,i*_ and *d*_*pred,i*_ represent experimental and calculated deposition values, respectively.

**Figure 9:**
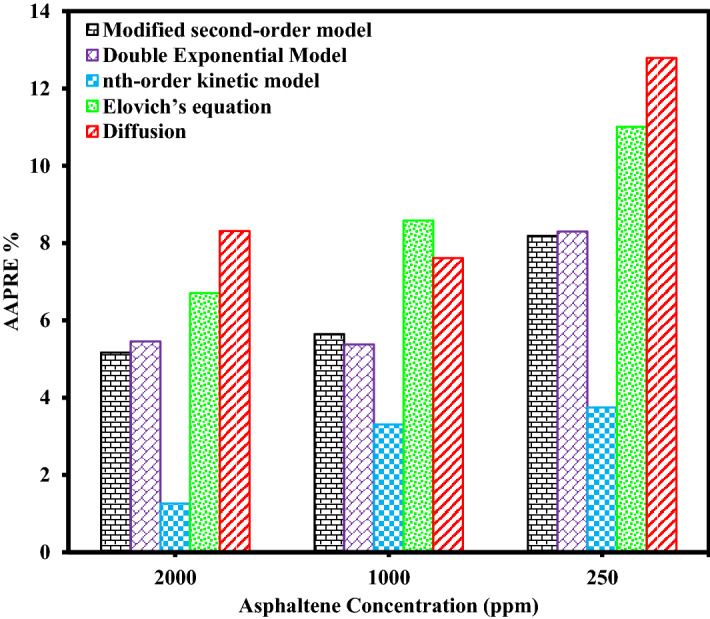
Comparison of deposition kinetics models at different asphaltene concentrations for PTFE coatings at static state based on AAPRE.

**Figure 10:**
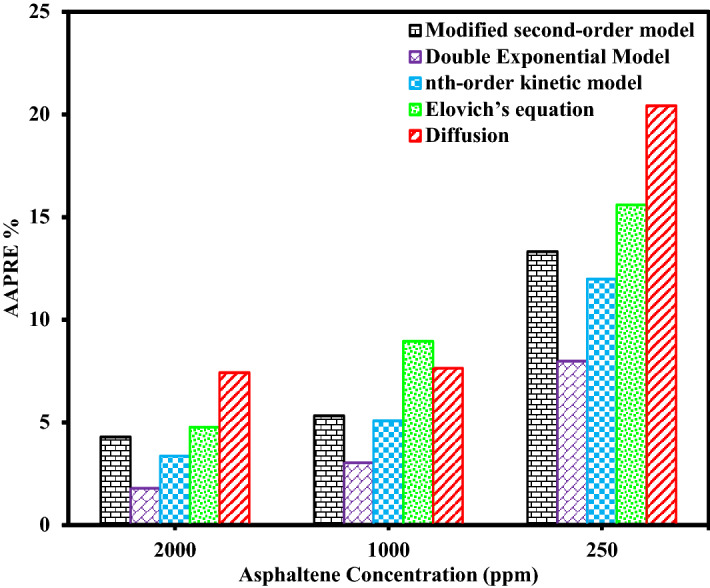
Comparison of deposition kinetics models at different asphaltene concentrations for PTFE coatings at dynamic state based on AAPRE.

**Figure 11:**
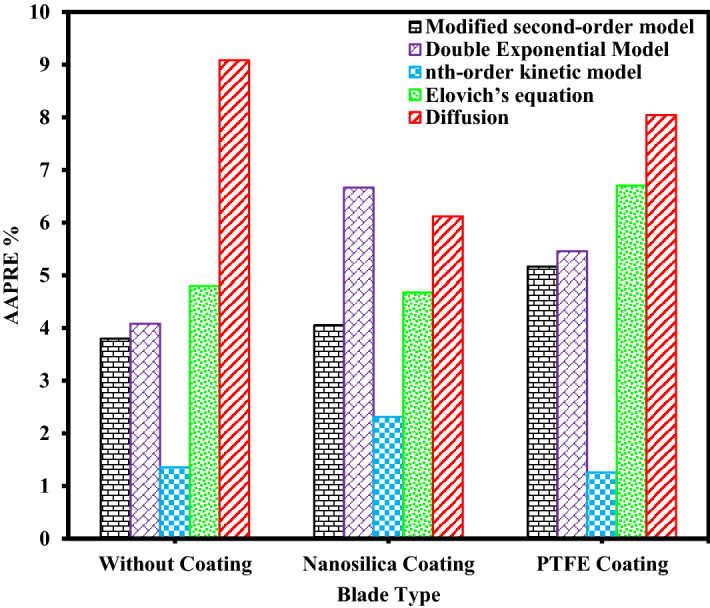
Comparison of deposition kinetics models for different coatings at concentration of 2000 ppm in static state based on AAPRE.

**Figure 12:**
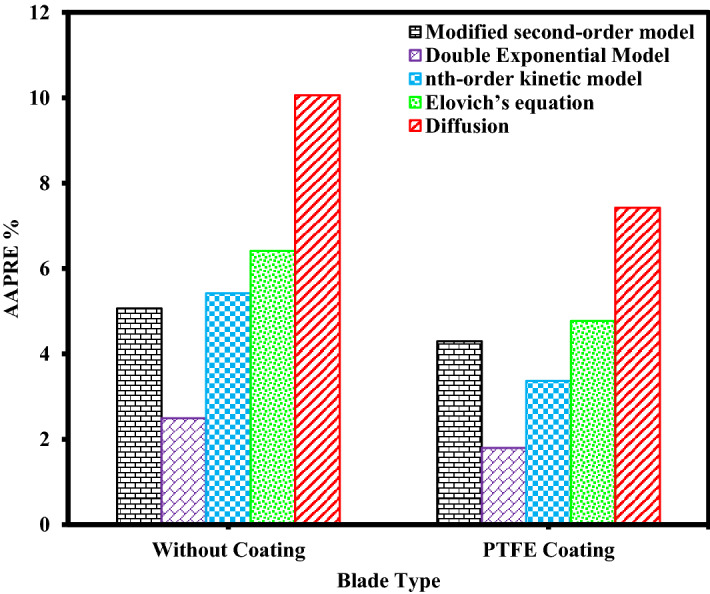
Comparison of deposition kinetics models for different coatings at concentration of 2000 ppm in dynamic state based on AAPRE.

**Figure 13:**
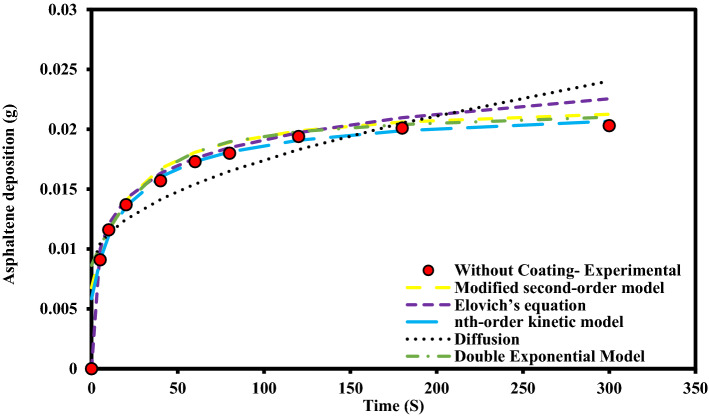
Comparison of experimental data and deposition kinetic models for uncoated electrode at 2000 ppm concentration in static state.

**Figure 14:**
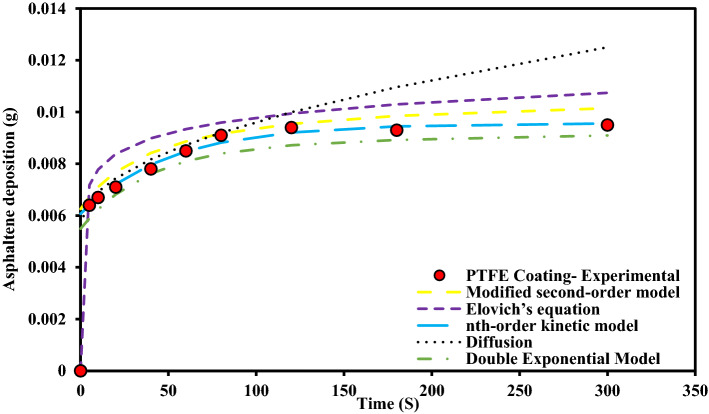
Comparison of experimental data and deposition kinetic models for PTFE coating electrode at 2000 ppm concentration in static state.

**Figure 15:**
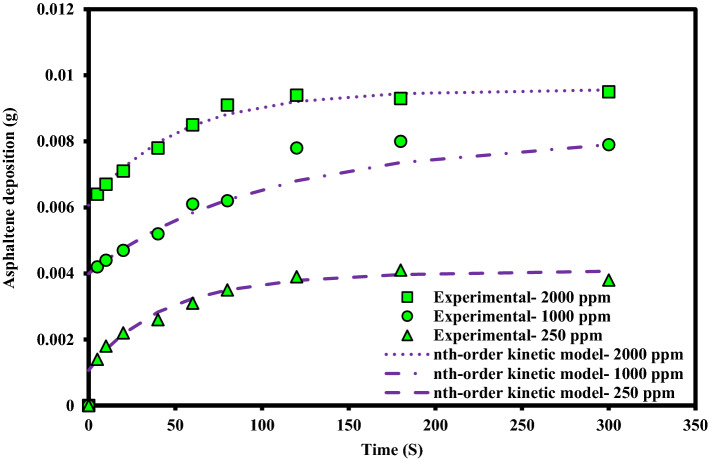
Agreement of the nth-Order deposition kinetic model to experimental data for different concentrations at static state for PTFE coating.

**Figure 16:**
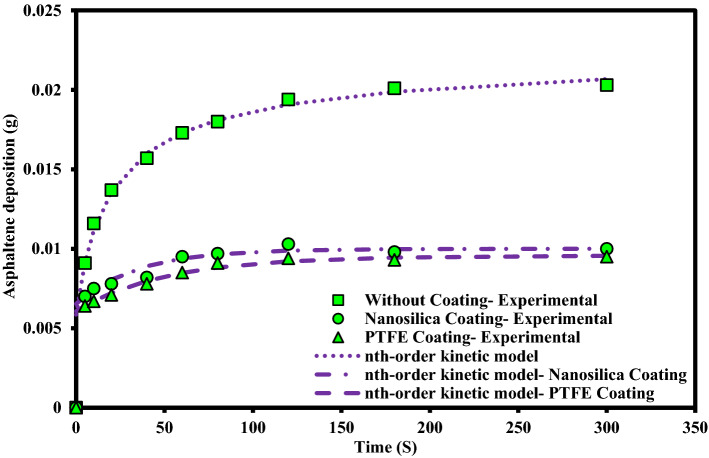
Agreement of the nth-Order deposition kinetic model to experimental data for different electrodes at static state.

The results of calculated *AAPRE* and *RMSE* parameters for all kinetic models evaluated in this study are listed in Table 9. Figures 9, 10, Supplementary Figs. S.2, and S.3 show the comparison of *AAPRE* and *RMSE* for deposition kinetics models at different concentrations of asphaltenes for PTFE coatings, in static and dynamic states. Also in Figs. 11, 12, Supplementary Figs. S.4, and S.5 the deposition kinetics models for different coatings at concentrations of 2000 ppm in static and dynamic states based on *AAPRE* and *RMSE* parameters are illustrated. Figures 13, 14, Supplementary Figs. S.6, and S.7 show the kinetic models that fit the experimental data for the uncoated electrode and the PTFE coated electrode at a concentration of 2000 ppm, in static and dynamic states. Figures 15 and 16 illustrate the agreement of the nth-order kinetics model to the experimental data in a static state for different concentrations and electrodes. Also, Supplementary Figs. S.8 and S.9 illustrate the agreement of the double exponential model to the experimental data in the dynamic state for different concentrations and electrodes. In all kinetic models, the calculated *Q*_*e*_ values are approximately equal to the experimental *Q*_*e*_ values.

**Figure 17:**
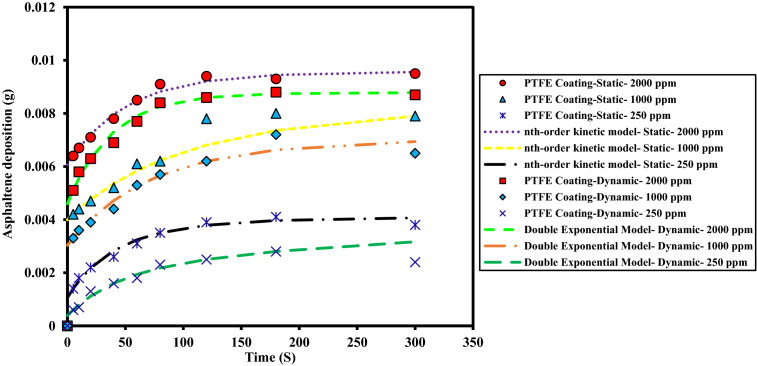
Overall comparison between experimental data and the best deposition kinetics models for PTFE coating electrodes at different concentrations in static and dynamic states.

As can be seen in these figures, the diffusion model and Elovich’s equation do not fit well with the experimental data and have more errors than the other ones. The selection of suitable deposition kinetics models is based on the deposition process mechanism and application of different models. Based on the deposition kinetics data, it can be concluded that at the beginning of the asphaltene deposition process, the maximum amount of deposition occurs on the electrode surface (during the first 2 min), and then the amount of deposition decreases. Also, based on these figures, it can be seen that the presence of a coating on the electrode surface caused a reduction of about 56% of the deposition in the static state at the concentration of 2000 ppm. Comparison of the amount of asphaltene deposition in the dynamic state for the coated electrodes shows more than 70% reduction in the amount of deposition at the same concentration, compared to the uncoated electrode. Regarding the *RMSE* values, in the static state, Elovich’s equation and based on *AAPRE* values, the nth-order kinetics model showed the best fit to the experimental data. For the dynamic state and based on the *RMSE* parameter, Elovich’s equation, has the best fit to the experimental data, and from *AAPRE* comparison, the double exponential model shows better agreement with the experimental data. Comparison of both parameters at all concentrations shows that in the static state, the nth-order kinetics model and in the dynamic state, the double exponential model, have the best agreement with the experimental data. Figure 17 shows the agreement of the experimental data and the best deposition kinetic models in static and dynamic states for PTFE coating electrodes at different concentrations.

In this study, the adsorption kinetic modeling showed that the initial deposition rate is faster than that at subsequent times. According to the literature, most unstable fractions of asphaltenes, which have a high metal content and are more polar, react to the electric field and form the first adsorbed layer on the electrode, which will lead to subsequent deposition^69–71^. Other studies have also shown that polar entities of asphaltene dominate the initial adsorption mechanism^64,72–74^. Therefore, it can be said that the first layer of asphaltene particles is bonded to the electrode surface under the influence of an electric field and starts a chemical reaction with it, but other particles are mainly affected by solvent interaction due to lack of direct contact with the electrode surface.

## Conclusions

The application of internal coatings with low surface energy could help to tackle organic and inorganic scale depositions in pipes. Recent efforts have been devoted to producing coatings with special surface properties to prevent or minimize asphaltene deposition on metal srfaces. In this study, two superhydrophobic coatings, including PTFE and nanosilica coatings, were fabricated simply and practically, and their performance for reducing asphaltene deposition was investigated. In this study, the effect of various factors including the type of coatings, fluid flow states, asphaltene concentration, and deposition time on the amount of asphaltene deposition was investigated and finally, the kinetics of asphaltene deposition in all these states were evaluated. We tried to show how the superhydrophobicity of a surface could increase its anti-scaling performance. Field application of this technique requires a comprehensive economic study based on net present value (NPV) analysis. This is an essential part of our future direction for extending the application of this technique in the field. The main findings of this study are as follows:

1. Surface wettability plays a major role in the amount of deposited asphaltene. Although both superhydrophobic coatings introduced in this study are capable of reducing the asphaltene deposits as compared to the uncoated electrode, the PTFE coating showed better performance.
2. At an asphaltene concentration of 2000 ppm and compared to the uncoated electrode the PTFE coated electrode shows a 56% decrease in asphaltene depositions at static state and more than 70% decrease in the number of asphaltene depositions at dynamic state.
3. The maximum amount of asphaltene deposition on the surface of the electrodes occurs during the first 120 s of the electrodeposition process.
4. The change of flow state affects the asphaltene deposition kinetics on the electrode surfaces. However, the type of electrode has no effect on the kinetics of asphaltene deposition. The results showed that the diffusion model and Elovich’s equation are not in good agreement with the experimental data and the highest error is observed in these two ones. Investigation of the effect of flow type on the kinetics of asphaltene deposition showed that in the static state, the nth-order kinetics model, and in the dynamic state, the double exponential model has the best agreement with the experimental data.

## Supplementary Information

Supplementary Information.
