# Supplementary material for: Experimental study and modelling of asphaltene deposition on metal surfaces with superhydrophobic and low sliding angle inner coatings
Source: Sci Rep. 2021 Aug 19;11:16812. doi: 10.1038/s41598-021-95657-5 (PMC8377080; doi:10.1038/s41598-021-95657-5)
Supplement: Supplementary file 1 — Supplementary Information. [file 41598_2021_95657_MOESM1_ESM.docx]

**Experimental study and modelling of asphaltene deposition on metal surfaces with superhydrophobic and low sliding angle inner coatings**

Mohammad Haji-Savameri ^a^, Saeid Norouzi-Apourvari ^a*^, Ahmad Irannejad ^b^, Abdolhossein Hemmati-Sarapardeh ^a, c, d,*^, Mahin Schaffie ^a^, Amir Mosavi ^e, f,*^

*^a^ Department of Petroleum Engineering, Shahid Bahonar University of Kerman, Kerman, Iran*

*^b^ Department of Materials Engineering and Metallurgy, Shahid Bahonar University of Kerman, Kerman, Iran*

*^c^ Institute of Research and Development, Duy Tan University, Da Nang 550000, Vietnam*

*^d^ Faculty of Environment and Chemical Engineering, Duy Tan University, Da Nang 550000, Vietnam*

*^e^ John von Neumann Faculty of Informatics, Obuda University, 1034 Budapest, Hungary*

**Supplementary File:**

**1. Evaluation of asphaltene deposition rate**

**1.1. Comparison of asphaltene deposition on uncoated and PTFE coated electrodes at a concentration of 250 ppm.**

|  |
| --- |
| **Fig. S.1.** Asphaltene deposition rate on uncoated and PTFE coated electrodes for different exposure times to electric field at ambient pressure and temperature (Asphaltene concentration 250 ppm, Electric field strength 2 kV/cm). |

**2. Evaluation of kinetic studies**

**2.1. Comparison of deposition kinetics models based on RMSE.**

|  |
| --- |
| **Fig. S.2.** Comparison of deposition kinetics models at different asphaltene concentrations for PTFE coatings at static state based on RMSE. |

|  |
| --- |
| **Fig. S.3.** Comparison of deposition kinetics models at different asphaltene concentrations for PTFE coatings at dynamic state based on RMSE. |

|  |
| --- |
| **Fig. S.4.** Comparison of deposition kinetics models for different coatings at concentration of 2000 ppm in static state based on RMSE. |

|  |
| --- |
| **Fig. S.5.** Comparison of deposition kinetics models for different coatings at concentration of 2000 ppm in dynamic state based on RMSE. |

**2.2. Comparison of experimental data and deposition kinetics models in dynamic state.**

|  |
| --- |
| **Fig. S.6.** Comparison of experimental data and deposition kinetics models for uncoated electrode at 2000 ppm concentration in dynamic state. |

|  |
| --- |
| **Fig. S.7.** Comparison of experimental data and deposition kinetics models for PTFE coating electrodes at 2000 ppm concentration in dynamic state. |

**2.3. Agreement of the double exponential kinetics model to experimental data in dynamic state.**

|  |
| --- |
| **Fig. S.8.** Agreement of the double exponential kinetics model to experimental data for different concentrations at dynamic state for PTFE coating. |

|  |
| --- |
| **Fig. S.9.** Agreement of the double exponential kinetics model to experimental data for different electrodes at dynamic state. |

**Table S.1.** Calculated AFM parameters for PTFE and nanosilica coatings.

| Type of Coating | RMS Rough | Ave Rough | Mean Ht |
| --- | --- | --- | --- |
| PTFE Coating | 1.255 µm | 972.1 nm | 2.926 µm |
| Nanosilica Coating | 611.2 nm | 539.8 nm | 1.014 µm |
